# Listening to an Audio Drama Activates Two Processing Networks, One for All Sounds, Another Exclusively for Speech

**DOI:** 10.1371/journal.pone.0064489

**Published:** 2013-05-29

**Authors:** Robert Boldt, Sanna Malinen, Mika Seppä, Pia Tikka, Petri Savolainen, Riitta Hari, Synnöve Carlson

**Affiliations:** 1 Brain Research Unit, O.V. Lounasmaa Laboratory, School of Science, Aalto University, Espoo, Finland; 2 Neuroscience Unit, Institute of Biomedicine/Physiology, University of Helsinki, Helsinki, Finland; 3 Advanced Magnetic Imaging Centre, O.V. Lounasmaa Laboratory, School of Science, Aalto University, Espoo, Finland; 4 Department of Film, Television and Scenography, School of Arts, Design and Architecture, Aalto University, Helsinki, Finland; 5 School of Medicine, University of Tampere, Tampere, Finland; Baycrest Hospital, Canada

## Abstract

Earlier studies have shown considerable intersubject synchronization of brain activity when subjects watch the same movie or listen to the same story. Here we investigated the across-subjects similarity of brain responses to speech and non-speech sounds in a continuous audio drama designed for blind people. Thirteen healthy adults listened for ∼19 min to the audio drama while their brain activity was measured with 3 T functional magnetic resonance imaging (fMRI). An intersubject-correlation (ISC) map, computed across the whole experiment to assess the stimulus-driven *extrinsic* brain network, indicated statistically significant ISC in temporal, frontal and parietal cortices, cingulate cortex, and amygdala. Group-level independent component (IC) analysis was used to parcel out the brain signals into functionally coupled networks, and the dependence of the ICs on external stimuli was tested by comparing them with the ISC map. This procedure revealed four extrinsic ICs of which two–covering non-overlapping areas of the auditory cortex–were modulated by both speech and non-speech sounds. The two other extrinsic ICs, one left-hemisphere-lateralized and the other right-hemisphere-lateralized, were speech-related and comprised the superior and middle temporal gyri, temporal poles, and the left angular and inferior orbital gyri. In areas of low ISC four ICs that were defined intrinsic fluctuated similarly as the time-courses of either the speech-sound-related or all-sounds-related extrinsic ICs. These ICs included the superior temporal gyrus, the anterior insula, and the frontal, parietal and midline occipital cortices. Taken together, substantial intersubject synchronization of cortical activity was observed in subjects listening to an audio drama, with results suggesting that speech is processed in two separate networks, one dedicated to the processing of speech sounds and the other to both speech and non-speech sounds.

## Introduction

In everyday life, we are surrounded by environmental sounds, speech, and music. Recent methodological advances with naturalistic settings in functional brain imaging have given new insight into how the brain copes with this kind of complex environment [1], [2], [3], [4], [5], [6], [7], [8], [9], [10]. For example, voxel-wise intersubject correlation (ISC) analysis of functional magnetic resonance imaging (fMRI) data collected from subjects freely viewing a movie demonstrated widespread intersubject synchronization of cortical activity on a time-scale of seconds [2]. In addition to this kind of stimulus-driven *extrinsic* brain activity, several cortical areas display *intrinsic* activity that is not usually correlated across subjects [11]. The intrinsic system is thought to be associated with inner-oriented processing [11], and thus less affected by the external input, except for general dampening during stimuli or tasks. Well-organized networks in the resting brain are sometimes referred to as intrinsic [12]. Our use of the terms *extrinsic* and *intrinsic* refers to the distinction between the extrinsic and intrinsic brain networks that was originally based on replicability of the within-subject activation during two movie-viewing sessions [11], [13]. During such a complex stimulus, the between-subjects temporal similarity of brain activity is typically high in extrinsic areas and low in intrinsic areas [11], [13], and the ISC map provides a good approximation of the stimulus-related extrinsic system.

Both extrinsic and intrinsic brain networks can be further characterized by independent component analysis (ICA) [14], [15]. ICA is a data-driven, blind signal separation method that uses higher-order statistics to estimate a predefined number of independent components (ICs). ICA can be used to explore subdivision of both resting state and stimulus-driven brain activity [16]. In fact, ICA conveys information of the spatially independent sub-networks within the ISC map [9].

A few earlier fMRI studies have focused on brain responses to naturalistic auditory stimulation by exposing the subjects to narratives read by a single person. ISC analysis of fMRI data collected from subjects listening to such a complex stimulus revealed synchronized activity in a wide network including the middle and superior temporal gyrus, anterior temporal lobe, superior and inferior frontal gyri, and cerebellum [5], [6], [8]. Furthermore, brain responses were organized into clusters with similar temporal receptive windows so that, for example, the early auditory cortices responded to short-time-scale changes in the auditory input while parietal and frontal areas had the longest temporal receptive windows, likely integrating information needed for comprehension of the full narrative [8].

Here we used fMRI to investigate the across-subjects similarity of brain responses to complex natural sounds presented in an audio drama, which contained dialogs, narrations, and natural non-speech sounds. An ISC map was computed to assess the across-subjects common features in the stimulus-driven extrinsic network. Additionally a group-level ICA was performed and the dependence of the ICs on external stimuli was tested by comparing them with the ISC map. As the extrinsic and intrinsic systems are, by definition, separate in their spatial extent and reactivity, the way external stimuli affect e.g. the default-mode network [10], [17] and likely also other parts of the intrinsic network is unclear. We thus explored the coupling of the extrinsic and intrinsic systems by examining the temporal correlations between the extrinsic and intrinsic ICs. A recent study [18] described two functional networks, one responding to a variety of auditory stimuli and another preferentially to speech. As our main focus was in the processing of auditory information in a naturalistic listening condition, we also aimed to classify brain activity as speech-sound-related, as non-speech-sound-related, or both.

## Materials and Methods

### 1. Subjects

Fifteen healthy right-handed young adults with no history of hearing problems, psychiatric or neurological illnesses participated in this study. One subject was excluded because of several instances of sudden translational head movement in the range of 6 mm. Another subject was excluded as he could not recall the storyline and failed to answer correctly to nine of eleven questions regarding the storyline. Thus, 13 subjects (6 females, 7 males; mean age 23.3 yrs, range 19–30 yrs) were included in the analysis.

The subjects were native Finns fluent in Finnish, and they participated after written informed consent. The study had received prior approval from the ethics committee of the Helsinki and Uusimaa Hospital District. Before the scanning, the subjects were screened according to the local MRI safety regulations.

### 2. Audio Drama Stimulus

The stimulus was an audio drama lasting for 18 min 51 s, comprising separate sequences extracted from a Finnish movie “Postia Pappi Jaakobille” (Letters to Father Jaakob, director Klaus Härö, Production company: Kinotar Oy, Finland, 2009).

In the movie, a woman released from jail arrives to a run-down parsonage where she is employed to help an old blind priest. Her main task is to read and write letters. The audio drama included sounds from the original movie, and an additional narration describing the visual actions and surroundings of the movie for blind people. The scenes from which the audio tracks were selected included a dialogue between a woman and a priest and between the priest and a mailman, as well as natural outdoor and indoor sounds, such as birdsong, rain, clatter of dishes, and sounds of furniture moved around. The movie was not shown at any stage of the experiment, but four subjects reported that they had previously seen it.

Before brain imaging, outside of the scanner, the subjects listened to a 9-min introduction in which the film director described the main characters and the scenery. This procedure guided all subjects to construct a similar understanding of the material before the experiment. During scanning, the stimulus was presented binaurally with an UNIDES ADU2a audio system (Unides Design, Helsinki, Finland) from a PC with an audio amplifier (Denon AVR-1802) and a power amplifier (Lab.gruppen iP 900). The sounds were delivered to the subject through plastic tubes connected to eartips (Etymotic Research, ER3, IL, USA) that were inserted into the ear canals. The subject’s hearing was further protected from the background noise of the magnet by earmuffs. Subjects were instructed to hold their eyes closed while they listened to the sound track.

A method-of-limits approach was used to obtain crude individual hearing thresholds. A complex 50-ms sound, comprising 5 sinusoidal tones of 300, 700, 1000, 1350, and 1850 Hz, was presented binaurally in descending and ascending series with 5 dB steps. The subject reported whether the sound was heard or not. The process was repeated until we found a threshold measure where the subject heard ≥70% of the sounds. The mean ± SD threshold intensity for the binaurally presented test sound was 10.4±2.9 dB SPL in each earplug. The sound level was first adjusted to 50 dB above the individual hearing threshold measured in the scanner room. We then replayed parts of the introduction to the subjects and gradually raised the sound level until the sound was loud but still comfortable to listen, resulting in an average sound level of 64 dB (range 60–70 dB) above the hearing threshold.

### 3. Constructing Sound Regressors

To model speech and non-speech sounds, we constructed a *speech regressor* (combined dialog and narration) and a *non-speech regressor* (all sounds excluding the dialog and narration), using Matlab-based MIRtoolbox (http://www.mathworks.com/matlabcentral/fileexchange/24583-mirtoolbox). We were granted access to the original movie soundtrack and thus had separate recordings for the dialogs, the narrations, and the surrounding sounds. The regressors were created by extracting, from the movie’s original multi-channel soundtracks, the dialog, and the narration into one soundtrack, and all other sounds into another soundtrack with a sampling rate of 48 kHz. Next a full-wave rectification and low-pass filtering (infinite impulse response filter with 8 Hz cut-off frequency) was performed. The smoothed signal was down-sampled with a factor of 16, resulting in a sampling rate of 3 kHz. The two regressors were convolved with a hemodynamic response function (HRF) and further down-sampled to 0.4 Hz to correspond to the sampling rate of the fMRI. The Matlab function used for resampling contained a linear-phase finite-impulse-response anti-aliasing filter.

The speech regressor contained, with minor exceptions, only speech; however, as the dialogs were not recorded in a studio, it also included some non-speech sounds related to acting (such as moving a chair). The non-speech regressor included music, echoes and other effects, such as birdsong and rain, and the stereophonically presented non-speech sounds sometimes elicited percepts of 3-D auditory space. Analysis of the root-mean-square (RMS) energy of the speech and non-speech soundtracks showed that speech was present for 60% and non-speech sounds for 65% of the audio drama. When speech and non-speech sounds occurred simultaneously, speech sounds were louder for 77% and non-speech sounds for 23% of the total time. On average, speech was 3.9 dB louder than the non-speech sounds.

### 4. fMRI Data Acquisition and Analysis

#### 4.1 Data acquisition and preprocessing

MRI data were obtained with a Signa VH/i 3.0 T MRI scanner (General Electric, Milwaukee, WI, USA). Functional images were acquired using a gradient echo-planar-imaging (EPI) sequence with the following parameters: TR = 2.5 s, TE = 30 ms, flip angle = 75°, FOV = 22.0 cm, matrix = 64×64, slice thickness = 3.5 mm, voxel size = 3.4×3.4×3.5 mm^3^ and number of oblique axial slices = 43. Altogether 462 volumes were collected, but 6 dummy volumes were automatically discarded. A structural image was acquired using a T1-weighted 3D-MPRAGE-sequence with TR = 10 ms, TE = 30 ms, preparation time = 300 ms, flip angle = 15°, FOV = 25.6 cm, matrix = 256×256, slice thickness = 1 mm, voxel size = 1×1×1 mm^3^, and number of axial slices = 178.

We first acquired the structural images. Next a 10-min resting-state scan was collected for other purposes, and we then collected the data for this experiment. The subjects were instructed to lie still with their eyes closed and to listen attentively to the audio drama. After the experiment we asked the subjects to fill a questionnaire in which they estimated their vigilance at the beginning, in the middle and at the end of the audio drama on a scale from 1–10 (1 = very sleepy, 10 = very attentive). They also answered eleven questions about the storyline and were asked to describe the main theme of the story with a few sentences.

To remove transient nonspecific signal changes related to the sudden onset and offset of the stimulus, we cut three EPI volumes from the start and two from the end of the experiment and, thus, 451 volumes were included in the analysis. The data were preprocessed using SPM8 software (http://www.fil.ion.ucl.ac.uk/spm/) by including realignment, co-registration of the functional images to the anatomical images, normalization into MNI space, and smoothing with a Gaussian filter (full-width-half-maximum 8 mm). No slice-time correction was performed.

#### 4.2 Intersubject correlation

For ISC calculation, the variance of movement (six realignment parameters from the SPM realignment procedure), linear drift, constant term, and the global mean signal were removed from the data through linear regression. Correlation images of all subject pairs were calculated. First the Pearson’s voxel-by-voxel correlation coefficients were determined and, then, Fisher transformation was applied to convert the correlation coefficients to normally distributed variables. Correlation images for all subject pairs were used to search for statistically significant correlations at group-level. T-statistics exceeding the threshold corresponding to p<0.01 (family-wise error [FWE] corrected, t >5.1, 77 degrees of freedom [df]) and the extent of 20 voxels were defined as brain areas with statistically significant correlations between subjects [9]. The df value 77 refers to the total number of subject pairs (n*(n–1)/2 = 13*12/2 = 78). A null distribution obtained with a Monte Carlo simulation, and a previous study [5] show this to be a valid df for the ISC analysis.

#### 4.3 General linear model

Analysis based on general linear model (GLM) was performed in SPM8 to assess brain areas related to the processing of either speech or non-speech sounds. The GLM design matrix included the speech and non-speech regressors, a linear drift model, and the global mean and realignment parameters. The correlation coefficient between the speech and non-speech regressors was 0.09 (p = 0.056). Serial correlations were handled with a first order autoregressive [AR(1)] model. The contrast images of the main effects from each subject (mean responses to either speech or non-speech regressor) were entered into second-level analysis and t-tests were performed. The resulting activity maps were thresholded at FWE-corrected p<0.05 and cluster size >10 voxels.

#### 4.4 Independent component analysis, and sorting of the independent components

Independent component analysis was performed with the group ICA toolbox GIFT v1.3 (http://icatb.sourceforge.net/). The minimum-description-length algorithm [19] implemented in GIFT estimated the mean number of sources to be 55. Spatial ICs were determined using the Infomax algorithm [20]. The ICASSO method [21] was used to assess the replicability of the ICs by running the algorithm 150 times. As the ICA typically gives slightly different results each time, the result was a cluster of estimates for each IC. The most representative IC of each cluster was selected as the IC of choice. Back-reconstruction of individual ICs and time-courses was done with GICA3 [22], also implemented in GIFT software. For labeling and visualization, the subject-specific images for each IC were entered into a second-level analysis and subjected to one-sample t-tests in SPM8 [4] with a FWE-corrected threshold of p<0.01 and cluster size >20 voxels.

The ISC map can be used as a functional template identifying extrinsic stimulus-related ICs [9]. To identify ICs with the strongest stimulus-related extrinsic characteristics, we sorted the ICs according to their spatial correlations with the thresholded ISC map using the GIFT software. As the method is biased by the spatial extent of the thresholded ICs, we additionally calculated the percent overlap of the ICs with the ISC map. An IC that had strong or moderate positive correlation (r >0.3) and >50% overlap with the ISC map was defined as an extrinsic IC. Because of these strict criteria, some extrinsic ICs may have been labeled as intrinsic.

Although the ICs derived from a spatial ICA are spatially maximally independent, their time-courses can exhibit temporal dependencies as has been demonstrated with both real and simulated data [23], [24]. Such temporal dependencies among spatial ICs can inform about functional network connectivity [25], and we thus searched for functional network connectivity by correlating–within each subject–the time-courses of the stimulus-related extrinsic ICs with the non-extrinsic ICs. For each pair of the extrinsic and intrinsic ICs the Pearson’s correlation coefficient was determined and, then, Fisher transformation was applied to convert the correlation coefficients to normally distributed variables. A two-tailed one-sample t-test (p<0.05, Bonferroni corrected for 160 tests, 12 df) was employed to identify significant correlations.

To describe the ICs according to their reactivity to speech and non-speech sounds, we subjected each IC time-course to a multiple regression analysis comparing them with the speech and non-speech regressors.

#### 4.5 Labeling of activation areas and visualizing the activations

Activations seen in the group analysis were labeled with the Automated Anatomical Labeling (AAL) tool [26]. Mricron (http://www.cabiatl.com/mricro/) and FreeSurfer (http://surfer.nmr.mgh.harvard.edu/) were used for visualization.

## Results

### 1. Vigilance Questionnaire

The mean ± SD vigilance reported by the subjects was 6.9±1.8 in the beginning, 5.5±1.8 in the middle, and 4.6±2.7 in the end of the experiment. Six subjects answered correctly to all eleven questions, six to ten questions, and one subject to nine. The subject discarded for drowsiness answered only two questions correctly.

### 2. Intersubject-correlation Map

**Figure 1:**
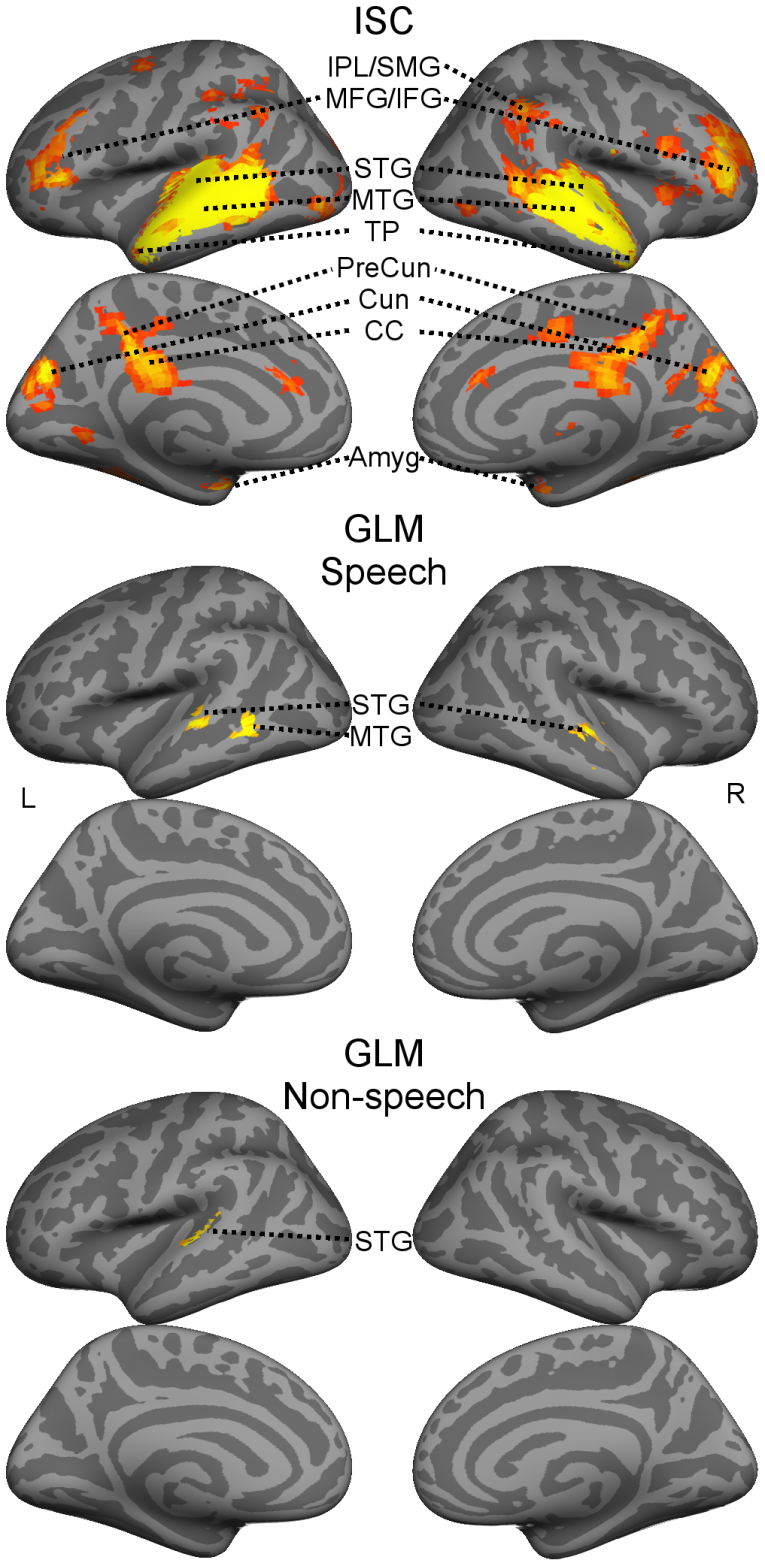
The ISC map and the GLM results for speech and non-speech sounds. The intersubject correlation (ISC) map (top panels) and general linear model (GLM) results overlaid on a MNI305 template brain and viewed from the lateral and medial aspects of both hemispheres. The GLM maps show brain areas correlating with the speech sounds (middle panels) and non-speech sounds (bottom panels). The GLM maps are thresholded at FWE-corrected p<0.05, cluster size >10 voxels, the ISC map at FWE corrected p<0.01, cluster size >20 voxels. STG = superior temporal gyrus, MTG = middle temporal gyrus, IPL = inferior parietal lobule, SMG = supramarginal gyrus, TP = temporal pole, MFG = middle frontal gyrus, IFG = inferior frontal gyrus, Cun = Cuneus, PreCun = PreCuneus, CC = cingulate cortex, Amyg = amygdala, L = left hemisphere, R = right hemisphere.

Figure 1 (top) shows the ISC map viewed from the lateral and medial aspects of both hemispheres. Six larger bilateral activation clusters are evident, covering the superior and middle temporal gyri (STG, MTG), extending to the temporal pole (TP), the middle/posterior cingulate cortex (CC), precuneus (PreCun), the cuneus (Cun), and the middle and inferior frontal gyrus (MFG/IFG). Further, bilateral activations were seen in the inferior parietal lobe and supramarginal gyrus (IPL/SMG) and amygdala (Amyg). Further activations (not labeled in the figure) were in the Heschl’s gyrus, Rolandic operculum, angular gyrus and hippocampus.

Moreover, unilateral activations were found in the left pre- and postcentral gyrus (Pre and PostCG), left lingual gyrus, left calcacrine gyrus, left superior and middle occipital gyrus, left cerebellum, right anterior CC, right supplementary motor area, and right fusiform gyrus; for more details, see Table 1.

**Table 1:** Peak voxel coordinates (x, y, and z in MNI system) and anatomical labels for the ISC map.

	x	y	z	Region	N
Left Hemisphere	–60	–18	2	Superior and middle temporal gyrus, Rolandic operculum, Heschl’s gyrus, †temporal pole, postcentral gyrus, supramarginal gyrus, angular gyrus	1168
	–12	–77	30	Cuneus, precuneus, superior occipital gyrus, calcacrine gyrus, lingual gyrus, cerebellum	467
	–46	35	10	Middle frontal gyrus, *frontal inferior triangular part	198
	–26	–4	–18	Amygdala, hippocampus	51
	–40	–77	2	Middle occipital gyrus	64
	–26	–7	48	Superior frontal gyrus, precentral gyrus	46
	–29	–42	–15	Fusiform gyrus	84
	–54	–52	48	Inferior parietal gyrus	28
	–40	–42	44	Inferior parietal gyrus	30
	–40	–24	44	Postcentral gyrus	22
Right Hemisphere	66	–14	2	Superior and middle temporal gyrus, Heschl’s gyrus, Rolandic operculum, †temporal pole,middle and posterior cingulate gyrus, precuneus, supplementary motor area,inferior parietal gyrus, supramarginal gyrus, angular gyrus	1544
	48	42	10	Middle and superior frontal gyrus, frontal inferior triangular and opercular part	440
	24	–7	–15	Amygdala, hippocampus	35
	2	35	24	Anterior cingulate gyrus	65
	27	–42	–15	Fusiform gyrus	28

* = rostral/anterior part.

† = posterior part.

N refers to the number of voxels in each cluster. Anatomical labeling is based on the group data, and was performed with the Automated Anatomical Labeling (AAL) tool. Labels are listed if an ISC map cluster extended ≥10 voxels into the AAL defined area.

### 3. Activations Related to Speech and Non-speech Sounds

Figure 1 (middle, bottom) also shows the GLM results for speech and non-speech sounds. Speech-related activations (middle panels) covered bilaterally the STG, and unilaterally the left MTG. The non-speech-related clusters (bottom panels) differed partly from the speech-related activations, covering only the left STG. Table 2 summarizes the activated areas. Since we used a naturalistic continuous stimulus, it was difficult to construct a proper set of GLM regressors describing specific stimulus features, which likely explains the limited activations found in the speech and non-speech GLM analyses. Thus data-driven methods (ICA and ISC) are beneficial for the analysis of brain activations related to this kind of stimuli.

**Table 2:** Peak voxel coordinates (x, y, and z in MNI system) and anatomical labels for the GLM maps.

	x	y	z	Region	N
Speech sounds regressor					
Left Hemisphere	–57	–46	6	Superior and middle temporal gyrus	72
Right hemisphere	66	–14	–1	Superior temporal gyrus	40
Non-speech sounds regressor					
Left hemisphere	–54	–24	16	Superior temporal gyrus	27

N refers to the number of voxels in each cluster. Anatomical labeling is based on the group data, and was performed with the Automated Anatomical Labeling (AAL) tool. Labels are listed if a GLM map cluster extended ≥10 voxels into the AAL defined area.

### 4. Independent Component Analysis

The ICA resulted in components involved in functionally plausible brain networks and other components possibly reflecting biological and non-biological noise sources. Eleven out of the original 55 ICs were discarded from further analysis: eight because of a stability index less than 0.9 (on a scale from 0 to 1) [21] and three that were not located in gray matter.

**Figure 2:**
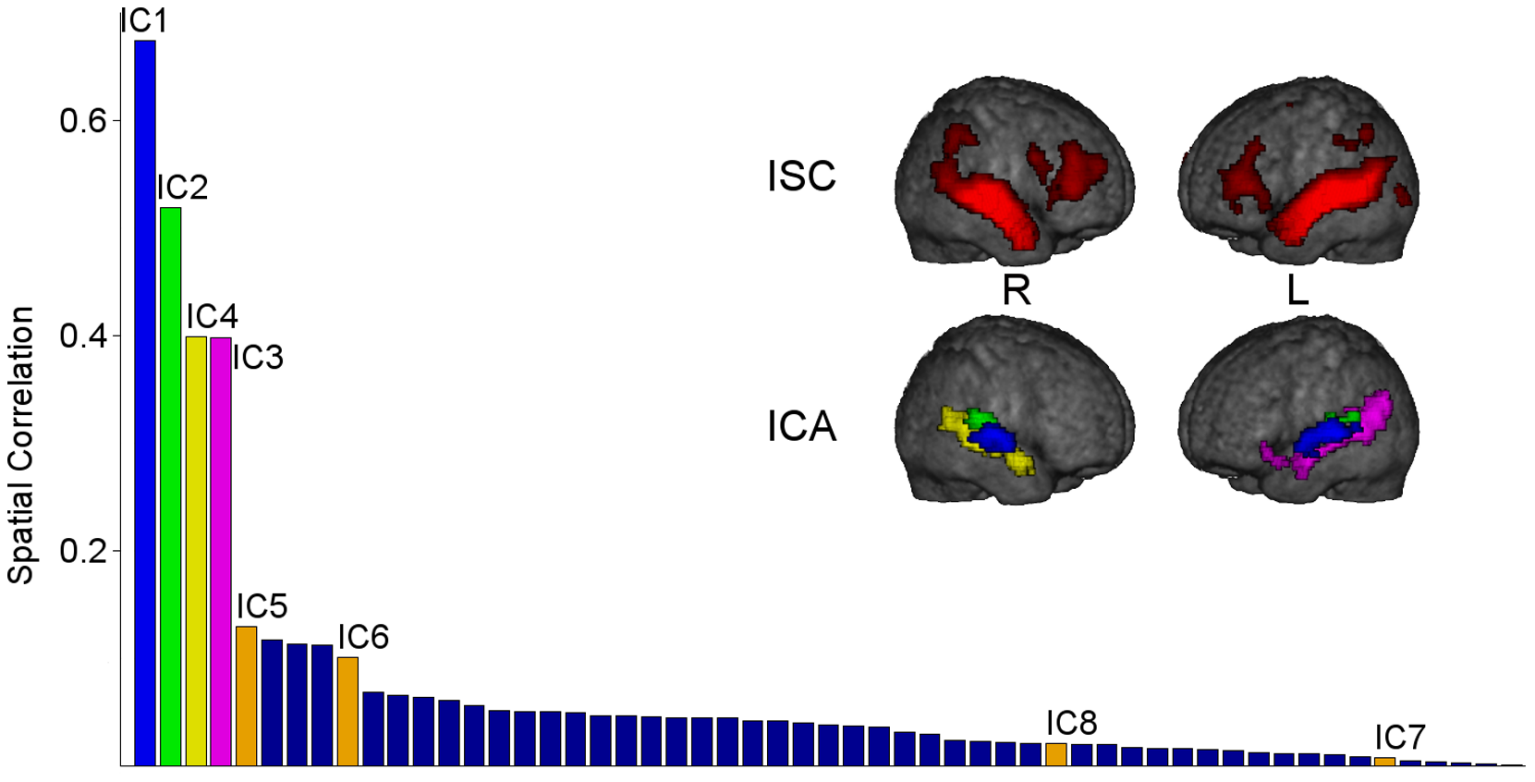
Sorting of the ICs based on correlations with the ISC map. The bar graph shows the independent components (ICs) organized in a descending order based on their spatial correlations with the intersubject correlation (ISC) map. The colors of the four most stimulus related extrinsic ICs (IC1–IC4) in the bar graph correspond to the colors in the independent component analysis (ICA) map illustrating their spatial distribution. The four ICs (IC5–IC8) that correlated temporally with two extrinsic ICs are also marked in the bar graph. The ISC and IC maps are thresholded at FWE corrected p<0.01, cluster size >20 voxels. L = left hemisphere, R = right hemisphere.

Figure 2 displays the spatial correlation coefficients of all 55 ICs with the ISC map in descending order, as well as the ISC map (top) and the ICA patterns for the four most correlated ICs (bottom). These four ICs (IC1–IC4) were defined as extrinsic. They had a strong spatial similarity with the ISC map with the following spatial correlations: IC1, r = 0.67; IC2, r = 0.52; IC3, r = 0.40; IC4, r = 0.40. Furthermore, of all ICs, IC1–IC4 had the highest percentage of overlap with the ISC map (overlaps 97%, 87%, 70% and 90%, for IC1–IC4, respectively). Table 3 lists the anatomical areas covered by these four extrinsic ICs and the coordinates of the peak voxel for each cluster.

**Table 3:** Peak voxel coordinates (x, y, and z in MNI system) and anatomical labels for IC1–IC8.

	x	y	z	Region	N
IC1	–60	–18	6	Superior and middle temporal gyrus	175
	62	–7	2	Superior temporal gyrus	130
IC2	–40	–21	16	Superior temporal gyrus, Rolandic operculum, Heschl’s gyrus, insula	267
	52	–21	10	Superior temporal gyrus, Rolandic operculum, Heschl’s gyrus, insula	248
IC3	–57	–46	13	Superior and middle temporal gyrus, angular gyrus	520
	–50	24	–12	†Temporal pole, inferior frontal gyrus orbital part	23
IC4	55	–32	–4	Superior and middle temporal gyrus, †temporal pole	419
IC5	–46	–4	–8	Superior temporal gyrus, insula, Rolandic operculum, ‡temporal pole, putamen	260
	48	14	–12	Superior temporal gyrus, insula, Rolandic operculum, ‡temporal pole, putamen	268
IC6	–57	–35	16	Supramarginal gyrus, superior temporal gyrus, inferior parietal gyrus	217
	66	–28	27	Supramarginal gyrus, superior temporal gyrus, Rolandic operculum	282
IC7*	2	56	27	Middle frontal gyrus, superior frontal gyrus, superior frontal gyrus medial part, supplementary motor area	568
IC8	2	–60	30	Precuneus, cuneus, calcacrine, middle and posterior cingulate cortex	538

* Left dominant.

† = Inferior/posterior part.

‡ = Superior/posterior part.

N refers to the number of voxels in each cluster. Anatomical labeling is based on the group data, and was performed with the Automated Anatomical Labeling (AAL) tool. Labels are listed if an IC cluster extended ≥10 voxels into the AAL defined area.

**Figure 3:**
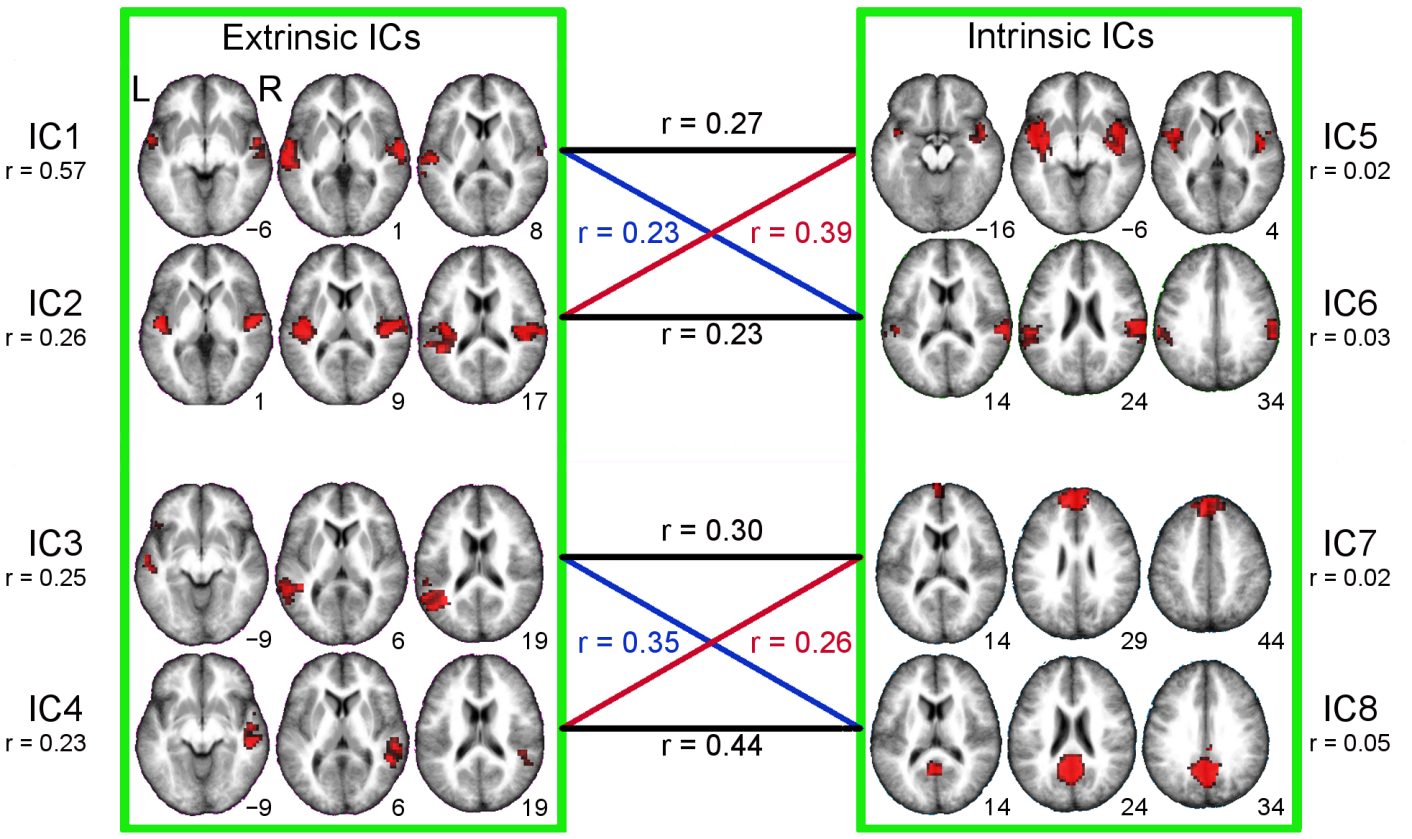
Spatial maps for IC1–IC8. The extrinsic independent components (ICs), IC1–IC4, are shown on the left and the intrinsic ICs, IC5–IC8 on the right. Extrinsic and intrinsic ICs are grouped with green boxes. Mean intersubject correlation values of the ICs time-courses are written below the IC number. Correlation values between the extrinsic and intrinsic ICs are displayed in the middle. The group-level t-maps are thresholded at FWE corrected p<0.01, cluster size >20 voxels and overlaid on an average of the subjects’ normalized anatomical images. MNI z-coordinates are presented beside the axial slices. L = left, R = right, r = mean Pearson correlation strength.

The time-courses of the four extrinsic ICs (IC1–IC4) correlated significantly (p<0.05, Bonferroni corrected) with the time-courses of 16 intrinsic ICs. The correlations were numerous, but here we describe correlations to the four intrinsic ICs (IC5–IC8, displayed in Figure 3) that seem linked to speech and non-speech sounds. Each of these four intrinsic ICs correlated significantly with two of the extrinsic ICs. The numbers in the middle of Figure 3 correspond to the mean correlation strengths between the extrinsic and intrinsic ICs.

**Figure 4:**
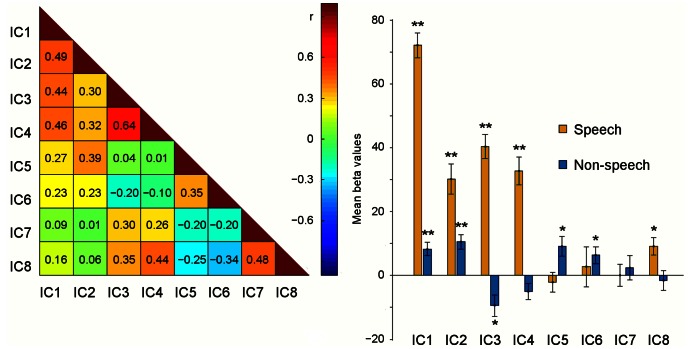
Correlations between the time-courses of the independent components (ICs) and reactivity of the ICs to speech and non-speech sounds. The correlation matrix for the time-courses of IC1–IC8 is presented on the left. The colour of each square represents the subjects’ mean correlation strengths. The colour key is given to the right of the matrix. The bar graph on the right presents mean beta value results from the analysis where the time-courses of the spatial ICs were subjected to a multiple regression analysis with the speech (brown) and non-speech (blue) regressors. * p<0.05, **p<0.005.

Figure 4 (left) shows the mean correlation strengths for the extrinsic IC1–IC4 and the intrinsic IC5–IC8 in a correlation matrix. The remaining 12 intrinsic ICs (IC9–IC20) that correlated significantly with the extrinsic ICs are described in supplementary materials (Figure S1 and Table S1 and S2). The time-courses of IC5 (encompassing bilaterally STG, Rolandic operculum, TP, putamen and insula) and IC6 (bilateral SMG, STG, left IPL) correlated significantly with the time-courses of the extrinsic IC1 and IC2, and the time-courses of IC7 (bilateral SFG and medial part of SFG, and right MFG) and IC8 (bilateral PreCun, Cun and posterior-CC) with those of the extrinsic IC3 and IC4. IC7 and IC8 encompassed areas of the medial prefrontal cortex, posterior-CC, and PreCun, i.e. areas that have been proposed to belong to the default-mode-network (DMN) [17]. One-way analysis of variance was used to test for differences in correlation strengths among the four extrinsic ICs towards each of IC5–IC8 (Table S3 and S4). Spatial correlations between these intrinsic ICs and the ISC map were low (Figure 2): IC5, r = 0.13; IC6, r = 0.10; IC7, r = 0.01; IC8, r = 0.02.

Table 3 lists the anatomical areas covered by ICs 1–8 and the coordinates of the peak voxel for each cluster.

Figure 4 (right) shows the activation strengths (beta values) for IC1–IC8 derived from a multiple regression analysis with both the speech and non-speech regressor (orange and blue colours, respectively). IC1–IC4 and IC8 fluctuated similarly as the speech regressor (IC1: partial r = 0.47, t (12) = 18.1, uncorrected p<0.0001; IC2: r = 0.22, p<0.0001; IC3: r = 0.28, p<0.0001; IC4: r = 0.23, p<0.0001; and IC8: r = 0.07, p = 0.0067, respectively). IC1, IC2, IC5 and IC6 correlated positively with the non-speech regressor (IC1: r = 0.10, t (12) = 3.7, p = 0.0026; IC2: r = 0.12, p = 0.0007; IC5: r = 0.14, p = 0.0130; IC6: r = 0.11, p = 0.0355, respectively). Negative correlations were found between IC3 and the non-speech sounds (r = –0.16, t (12) = –2.84, p = 0.0148).

## Discussion

We investigated the across-subjects similarity of brain activity to speech and non-speech sounds within an audio drama. Intersubject synchronization was strongest in the temporal lobe auditory areas, frontal cortex, and in the Cun and PreCun. The fMRI data were also analyzed using ICA, and by comparing the spatial overlaps of ICs with the ISC map, four ICs were determined extrinsic. These extrinsic ICs were modulated by either speech sounds or by both speech and non-speech sounds. ICs with little or no overlap with the ISC map were considered intrinsic.

Statistically significant functional connectivity was found between the stimulus-related extrinsic brain areas (MTG, STG, posterior insula, inferior TP, and MFG/IFG) and networks that were considered intrinsic (anterior insula, superior TP, SFG, middle- and posterior-CC, Cun, PreCun, and SMG/IPL). The time-courses of the intrinsic ICs correlated either with the time-courses of the extrinsic ICs reacting to the speech sounds or with those fluctuating similarly with speech and non-speech sounds.

**Figure 5:**
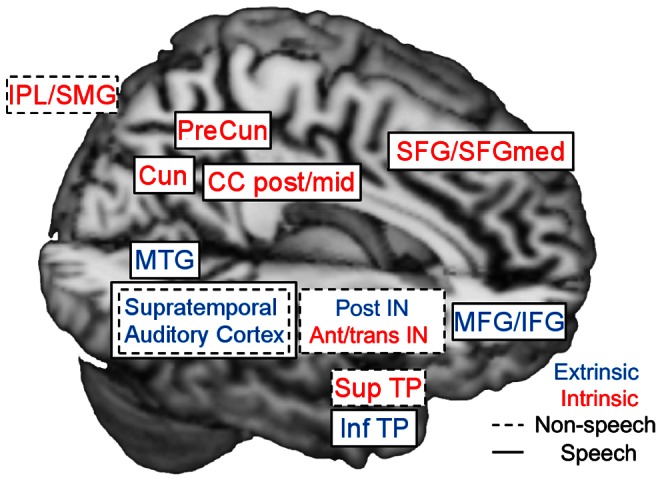
A schematic summary of the extrinsic and intrinsic speech and non-speech networks. The extrinsic areas are labeled with blue letters, the intrinsic with red letters. Areas of the speech network are marked by solid boxes, areas of the non-speech sound network by dotted boxes. The summary is based on the characteristics of independent components (ICs) IC1–IC8. Extrinsic areas are based on the correlation and overlap of the ICs with the intersubject correlation (ISC) map. Speech and non-speech brain areas are based on the similarity of the time-courses of the ICs to the speech and non-speech regressors, and on functional connectivity analysis. Abbreviations: MTG = middle temporal gyrus, IN = insula, SFG = superior frontal gyrus, SFGmed = superior frontal gyrus medial part, MFG = middle frontal gyrus, IFG = inferior frontal gyrus, TP = temporal pole, IPL = inferior parietal lobule, SMG = supramarginal gyrus, CC = cingulate cortex, Cun = Cuneus, PreCun = PreCuneus, ant = anterior, post = posterior, sup = superior, inf = inferior, trans = transitional.

We thus propose in Figure 5 the involvement of two separate large-scale networks in the processing of an audio drama: one best described as a speech network (boxes surrounded by solid lines), the other reacting to speech sounds and non-speech sounds (boxes surrounded by dashed lines). These networks only share the supratemporal auditory cortex and contain several nodes of both extrinsic (blue lettering) and intrinsic (red lettering) networks.

### 1. Extrinsic Independent Components

The ISC analysis revealed across-subjects synchronized activity in the Heschl’s gyrus, STG, MTG, and MFG/IFG, i.e. in cortical areas that previous ISC analysis experiments have linked to the processing of complex auditory stimuli [5], [6]. Across-subjects synchronized activity was also observed in the amygdala. As the audio drama consisted of several voices with strong acted emotions, the between-subjects synchronization in the amygdala might reflect responses to the emotional prosody of the actors [27], [28]. Accordingly, in an earlier study about brain responses to vocal expressions of anger and happiness, the amygdala responded to emotional vocal expression [29]. Interestingly, we also found ISC in the inferior TP, an area that is interconnected with the amygdala and is critical for linking the representation of faces, and possibly auditory cues, to person-specific memories [30]. Thus, it is possible that the intersubject synchronization of the amygdala [28] and TP reflect processing important for identifying speakers and voice prosody.

We identified four ICs that covered areas that were also displayed in the ISC map. Two of these extrinsic ICs, one with bilateral activity in the STG and the other with bilateral activity in the STG, Rolandic operculum, Heschl’s gyrus, and posterior insula, had time courses that varied similarly with the speech and non-speech sounds. The partial correlations between these extrinsic ICs and the speech regressor were larger than those for the non-speech regressor. The speech sounds of the audio drama were in the foreground making it easy to follow the dialogs and narrations. Accordingly, the hemodynamic fluctuations in the speech and non-speech-related ICs were mainly driven by the speech sounds. The third extrinsic IC, a left-lateralized extrinsic IC with activation in the MTG, STG, angular gyrus, inferior TP, and orbital part of IFG, correlated positively with the speech sounds and negatively with the non-speech sounds. An IC with a similar spatial distribution, as this left-lateralized extrinsic IC, was also previously characterized as extrinsic and sensitive to speech [9]. The fourth extrinsic IC with activity unilaterally in the right STG, MTG, and inferior TP correlated with the speech sounds but not with the non-speech sounds.

An earlier fMRI–ICA study exploring brain activity to 30-s story narrations presented in a block design to 5**–**19 year-olds [31] described six stimulus-related ICs of which four were similar to the extrinsic ICs we found. Two ICs of that study, partitioning the STG similarly as our speech and non-speech ICs, were suggested to participate in spectral and temporal processing of sounds. A left-lateralized network, comprising the MTG and IFG, resembling our left-lateralized speech-related IC, was suggested to integrate contents of sentences to larger narratives and integrate semantic, syntactic, and pragmatic information. Furthermore, a right-dominant IC found bilaterally in the STG and thought to be important for achieving a final interpretation of the story [31], was similar to our speech-related right-lateralized IC.

In fMRI studies, susceptibility artifacts in the amygdala and limbic forebrain regions can reduce the signal-to-noise ratio and increase between-subject variance [32], thereby possibly reducing the inter- and intra-subject correlations in the amygdala. However, one extrinsic IC correlated significantly with an IC encompassing the amygdala/hippocampal area, an area previously linked to narrative processing [31]. This amygdala/hippocampal IC overlapped the amygdala activation in the ISC map (Figure S1, IC18; Table S1). As a majority of the 78 individual pair-wise correlations were significant in the left and right amygdala cluster, and susceptibility artifacts in the amygdala increase intersubject variance [32], the result suggests that all subjects had a reasonable signal from the amygdala area.

### 2. Functional Network Connectivity

Correlation patterns in fMRI experiments might have non-neural causes such as head movement [33], or in the case of functional network connectivity, spatial overlap of true sources [22]. As the intrinsic ICs we focused on showed significant correlations with two extrinsic ICs, the risk of correlations due to various sources of confound was possibly reduced. Two intrinsic ICs correlated with the extrinsic speech-related ICs. Two other intrinsic ICs correlated with the extrinsic speech- and non-speech-related ICs. The latter intrinsic ICs fluctuated similarly as the non-speech regressor, and did not correlate with the speech regressor or the extrinsic speech ICs. The first of these non-speech-sounds sensitive intrinsic ICs (IC5) covered areas of the STG, Rolandic operculum, superior TP, and putamen and the border of the anterior and posterior insula. A functional connectivity study suggests that the insula could be partitioned in an anterior, posterior, and transitional zone [34]. The posterior insula is involved in sensory-motor integration [35], the anterior insula participates in the processing of emotions and belongs to the task-set system [35], [36], a system important for shifting attention, and the transitional zone might guide both attention and sensory motor integration [34]. The transitional insula activation to non-speech sounds seems plausible as non-speech sounds, such as foot steps, give a feeling of movement, and sounds such as birdsong can change the listener’s thought frame from an indoors scene to an outdoors scene. Localizing moving sounds is thought to involve the SMG [37], suggesting that the other intrinsic non-speech-sounds component, IC6, encompassing SMG is involved in spatial orientation based on auditory cues. Auditory orientation cues were found among the non-speech sounds.

We found that two extrinsic speech-related ICs correlated positively with two intrinsic ICs, one encompassing the MFG, SFG, and medial part of SFG and the other encompassing PreCun, Cun, and middle- and posterior-CC, i.e. areas that are attributed to the DMN [17]. The functional network connectivity between these intrinsic ICs encompassing DMN areas and the extrinsic speech ICs suggests that some DMN areas contribute indirectly to the perceptual experience that an audio drama, especially its speech, produces. Some ISC studies link PreCun, posterior-CC, and MFG areas to semantic processing [5] and speculate that PreCun and MFG integrate speech information over a timescale of more than 30 s [8]. Furthermore, an fMRI study in which subjects listened to words revealed connectivity between ICs comprising temporal, parietal, and frontal areas [38].

The extrinsic ICs had a strong relationship to the stimulus, while the intrinsic ICs had weak or no relationship to the stimulus. The extrinsic ICs covered areas corresponding to the previously described extrinsic cortical division [11]. The intrinsic ICs that we found in the inferior parietal cortices, posterior-CC, and PreCun correspond to the previously described intrinsic cortical division [11]. However, one intrinsic non-speech IC (IC5; the STG, insula, Rolandic operculum, and TP) encompassed areas that were earlier described as extrinsic. Taking into account the strict criteria used for defining extrinsic ICs, these areas might have both extrinsic and intrinsic characteristics.

A two-stream hypothesis of visual perception, describing a ventral stream that is involved in object identification and a dorsal stream that is involved in the guiding of vision for action and in recognizing object position, is commonly accepted [39], [40]. A similar dual-pathway model has been suggested for the auditory system, with two largely segregated processing streams, a ventral stream for the identification of objects based on audition, including speech and environmental sounds, and a dorsal stream for the localization of sounds in space [41], [42], [43], [44]. The dorsal stream might in addition have a role in speech perception and auditory-motor integration [45]. Although our current experimental setup was not specifically planned to explore the ventral and dorsal stream divisions, the present findings lend some support to the dual-stream model of auditory/speech processing (see Fig. 5). The extrinsic speech- and non-speech related network encompassed temporal cortical areas that are part of the ventral stream bordering the dorsal stream areas. The speech-related network covered areas of both the ventral and dorsal stream.

### Conclusion

In conclusion, the results showed substantial synchronization of cortical activity among the subjects listening to an audio drama and suggest that natural sounds are processed in two separate networks, one dedicated to speech processing and the other to both speech and non-speech sounds. This dual network division of auditory perception is suggested by data driven methods.

## Supporting Information

Figure S1 **IC9–IC20 depicted in three orthogonal directions.** These ICs had time-courses that correlated positively significantly with one of the extrinsic ICs (opposed to IC5–IC8 that correlated positively with two of the extrinsic ICs). L = left, R = right, A = anterior, P = posterior.
(TIFF)

Table S1 **The means of the Pearson Correlation values for the time-courses of IC1–IC4 towards the time-courses of IC9–IC20.**
(DOC)

Table S2 **Peak voxel coordinates (x, y, and z in MNI system) and anatomical labels for IC9–IC20.**
(DOC)

Table S3 **The four separate one-way ANOVAs comparing correlation strengths between the extrinsic components (IC1−IC4) time-courses towards each of the intrinsic components (IC5−IC8) time-courses.**
(DOC)

Table S4 **Tukey HSD (honestly significant difference) test for post hoc comparisons of correlation strengths between the extrinsic and intrinsic ICs time-courses listed separately for IC5–IC8.**
(DOC)
