# Supplementary figures and images for: Listening to an Audio Drama Activates Two Processing Networks, One for All Sounds, Another Exclusively for Speech
Source: PLoS One. 2013 May 29;8(5):e64489. doi: 10.1371/journal.pone.0064489 (PMC3667190; doi:10.1371/journal.pone.0064489)

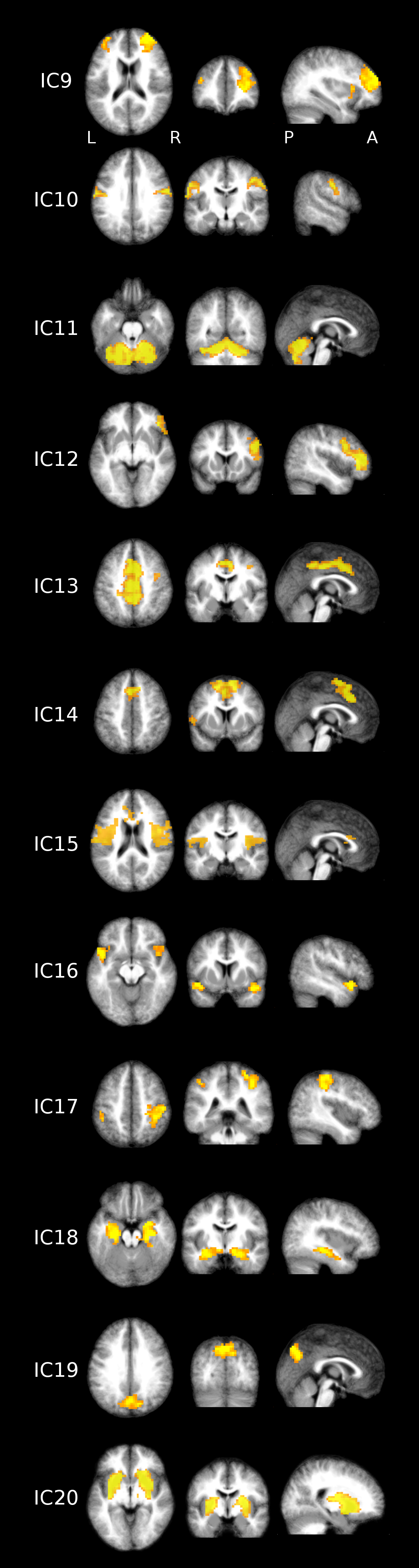

Supplement: Figure S1 — IC9–IC20 depicted in three orthogonal directions. These ICs had time-courses that correlated positively significantly with one of the extrinsic ICs (opposed to IC5–IC8 that correlated positively with two of the extrinsic ICs). L = left, R = right, A = anterior, P = posterior. (TIFF) [file pone.0064489.s001.tiff]
