# Supplementary material for: Listening to an Audio Drama Activates Two Processing Networks, One for All Sounds, Another Exclusively for Speech
Source: PLoS One. 2013 May 29;8(5):e64489. doi: 10.1371/journal.pone.0064489 (PMC3667190; doi:10.1371/journal.pone.0064489)
Supplement: Table S1 — The means of the Pearson Correlation values for the time-courses of IC1–IC4 towards the time-courses of IC9–IC20. (DOC) [file pone.0064489.s002.doc]

Table S1. The means of the Pearson Correlation values for the time-courses of IC1─IC4 towards the time-courses of IC9─IC20.

|  | IC9 | IC10 | IC11 | IC12 | IC13 | IC14 | IC15 | IC16 | IC17 | IC18 | IC19 | IC20 |
| --- | --- | --- | --- | --- | --- | --- | --- | --- | --- | --- | --- | --- |
| IC1 | −.04 | .13 | .29* | .10 | .14 | .23* | .12 | .19* | .01 | .20* | .05 | 0.16* |
| IC2 | .01 | .23* | .13 | .06 | .24* | −.02 | .39* | .09 | −.02 | .08 | .01 | 0.16 |
| IC3 | −.34* | −.06 | .06 | −.01 | −.21* | .00 | .04 | .12 | −.28* | .16 | −.22* | −0.02 |
| IC4 | −.17 | −.04 | .13 | .27* | −.09 | .09 | −.03 | .12 | −.20 | .19 | −.09 | 0.02 |

*p < 0.05, Bonferroni corrected
