# Supplementary material for: Listening to an Audio Drama Activates Two Processing Networks, One for All Sounds, Another Exclusively for Speech
Source: PLoS One. 2013 May 29;8(5):e64489. doi: 10.1371/journal.pone.0064489 (PMC3667190; doi:10.1371/journal.pone.0064489)
Supplement: Table S2 — Peak voxel coordinates (x, y, and z in MNI system) and anatomical labels for IC9–IC20. (DOC) [file pone.0064489.s003.doc]

Table S2. Peak voxel coordinates (x, y, and z in MNI system) and anatomical labels for IC9─IC20.

|  | x | y | z | Region | N |
| --- | --- | --- | --- | --- | --- |
| IC9 | 27 | 52 | 13 | Middle frontal gyrus, superior frontal gyrus, inferior frontal gyrus triangular part, insula | 481 |
|  | −32 | 52 | 16 | Middle frontal gyrus | 49 |
| IC10 | −64 | −14 | 24 | Postcentral gyrus | 105 |
|  | 58 | −10 | 34 | Postcentral gyrus, precentral gyrus | 126 |
| IC11 | −1 | −70 | −18 | Cerebellum, Vermis, Fusiform | 1640 |
| IC12 | 48 | 38 | 13 | Middle frontal gyrus, inferior frontal gyrus triangular, opercular and orbital part, precentral gyrus | 509 |
| IC13 | 6 | −24 | 44 | Middle cingulate cortex, supplementary motor area, precuneus, | 655 |
|  | 30 | −18 | 41 | Precentral gyrus | 20 |
| IC14 | 2 | 18 | 38 | Supplementary motor area, superior frontal gyrus, superior frontal gyrus medial orbital, middle frontal gyrus, middle cingulate cortex, precentral gyrus, | 634 |
|  | −54 | 18 | −1 | Inferior frontal gyrus opercular part | 31 |
| IC15 | −36 | 0 | 13 | Rolandic operculum, postentral gyrus, insula, inferior frontal gyrus opercular part, precentral gyrus, superior temporal gyrus | 337 |
|  | 44 | 0 | 20 | Rolandic operculum, insula, supramarginal gyrus, post and precentral gyrus | 405 |
|  | −12 | 35 | 16 | Anterior cingulate cortex | 50 |
| IC16 | −46 | 14 | −18 | Temporal pole | 96 |
|  | 44 | 7 | −22 | Temporal pole, insula | 93 |
| IC17 | 38 | −42 | 44 | Postcentral gyrus, supramarginal gyrus, inferior and superior parietal lobe, precentral gyrus | 412 |
|  | −43 | −46 | 48 | Inferior parietal lobe | 31 |
| IC18 | 34 | −24 | −18 | Hippocampus, parahippocampal gyrus, fusiform gyrus, amygdala, lingual gyrus, inferior temporal gyrus | 1069 |
| IC19 | 2 | −80 | 44 | Cuneus, precuneus, superior occipital lobe | 299 |
| IC20 | 27 | 1 | -1 | Putamen, caudate, insula, pallidum, thalamus, amygdala | 706 |
|  | -18 | 10 | 10 | Putamen, caudate, insula, pallidum, | 647 |

N refers to the number of voxels in each cluster. Anatomical labeling is based on the group data, and labeled with the Automatic Anatomical Labeling (AAL) tool. Labels are listed if an IC cluster extends more than 10 voxels into one of the AAL defined anatomical volumes of interest.
