# Supplementary material for: Listening to an Audio Drama Activates Two Processing Networks, One for All Sounds, Another Exclusively for Speech
Source: PLoS One. 2013 May 29;8(5):e64489. doi: 10.1371/journal.pone.0064489 (PMC3667190; doi:10.1371/journal.pone.0064489)
Supplement: Table S3 — The four separate one-way ANOVAs comparing correlation strengths between the extrinsic components (IC1−IC4) time-courses towards each of the intrinsic components (IC5−IC8) time-courses. (DOC) [file pone.0064489.s004.doc]

Table S3. The four separate one-way ANOVAs comparing correlation strengths between the extrinsic components (IC1−IC4) time-courses towards each of the intrinsic components (IC5−IC8) time-courses.

|  |  | Sum of Squares | df | Mean Square | F |
| --- | --- | --- | --- | --- | --- |
| IC5 | Correlation value variance between the extrinsic ICs | 0.56 | 3 | 0.19 | 16.9** |
|  | Correlation value variance within the extrinsic ICs | 0.53 | 48 | 0.01 |  |
|  | Total | 1.09 | 51 |  |  |
| IC6 | Correlation value variance between the extrinsic ICs | 0.53 | 3 | 0.18 | 27.4** |
|  | Correlation value variance within the extrinsic ICs | 0.31 | 48 | 0.01 |  |
|  | Total | 0.85 | 51 |  |  |
| IC7 | Correlation value variance between the extrinsic ICs | 0.30 | 3 | 0.10 | 9.10** |
|  | Correlation value variance within the extrinsic ICs | 0.52 | 48 | 0.01 |  |
|  | Total | 0.82 | 51 |  |  |
| IC8 | Correlation value variance between the extrinsic ICs | 0.57 | 3 | 0.19 | 24.5** |
|  | Correlation value variance within the extrinsic ICs | 0.37 | 48 | 0.01 |  |
|  | Total | 0.94 | 51 |  |  |

**p < 0.005

Degrees of freedom (df).
