# Supplementary material for: Listening to an Audio Drama Activates Two Processing Networks, One for All Sounds, Another Exclusively for Speech
Source: PLoS One. 2013 May 29;8(5):e64489. doi: 10.1371/journal.pone.0064489 (PMC3667190; doi:10.1371/journal.pone.0064489)
Supplement: Table S4 — Tukey HSD (honestly significant difference) test for post hoc comparisons of correlation strengths between the extrinsic and intrinsic ICs time-courses listed separately for IC5–IC8. (DOC) [file pone.0064489.s005.doc]

Table S4. Tukey HSD (honestly significant difference) test for post hoc comparisons of correlation strengths between the extrinsic and intrinsic ICs time-courses listed separately for IC5─IC8

|  |  |  |  | 95% CI | |
| --- | --- | --- | --- | --- | --- |
|  | Comparison | Mean Difference between correlations | Std.  Error | Lower Bound | Upper Bound |
| IC5 | IC1 vs. IC2 | −0.1 | 0.03 | −0.21 | 0.01 |
|  | IC1 vs. IC3 | 0.15* | 0.03 | 0.04 | 0.26 |
|  | IC1 vs. IC4 | 0.13* | 0.03 | 0.02 | 0.24 |
|  | IC2 vs. IC3 | 0.26* | 0.03 | 0.15 | 0.37 |
|  | IC2 vs. IC4 | 0.23* | 0.03 | 0.12 | 0.34 |
|  | IC3 vs. IC4 | −0.02 | 0.03 | −0.13 | 0.09 |
| IC6 | IC1 vs. IC2 | 0.01 | 0.02 | −0.08 | 0.09 |
|  | IC1 vs. IC3 | 0.18* | 0.02 | 0.10 | 0.26 |
|  | IC1 vs. IC4 | 0.23* | 0.02 | 0.14 | 0.31 |
|  | IC2 vs. IC3 | 0.17* | 0.02 | 0.09 | 0.26 |
|  | IC2 vs. IC4 | 0.22* | 0.02 | 0.14 | 0.30 |
|  | IC3 vs. IC4 | 0.05 | 0.02 | −0.04 | 0.13 |
| IC7 | IC1 vs. IC2 | 0.05 | 0.03 | −0.06 | 0.16 |
|  | IC1 vs. IC3 | −0.11† | 0.03 | −0.21 | 0.00 |
|  | IC1 vs. IC4 | −0.14* | 0.03 | −0.25 | −0.03 |
|  | IC2 vs. IC3 | −0.15* | 0.03 | −0.26 | −0.04 |
|  | IC2 vs. IC4 | −0.18* | 0.03 | −0.29 | −0.08 |
|  | IC3 vs. IC4 | −0.03 | 0.03 | −0.14 | 0.08 |
| IC8 | IC1 vs. IC2 | 0.06 | 0.02 | −0.03 | 0.15 |
|  | IC1 vs. IC3 | −0.21* | 0.02 | −0.30 | −0.11 |
|  | IC1 vs. IC4 | −0.13* | 0.02 | −0.22 | −0.04 |
|  | IC2 vs. IC3 | −0.27* | 0.02 | −0.36 | −0.18 |
|  | IC2 vs. IC4 | −0.19* | 0.02 | −0.28 | −0.10 |
|  | IC3 vs. IC4 | 0.08 | 0.02 | −0.01 | 0.17 |

* p < 0.05, † p = 0.059
